# Thyme Essential Oil Nanoemulsion Stabilized by Chitosan Nanoparticles for Potential Application in Food Preservation

**DOI:** 10.3390/polym18091012

**Published:** 2026-04-22

**Authors:** Lindoval S. Fonseca, Marcos A. das Neves, Mitsutoshi Nakajima, Barbara C. Damasceno, Lívia A. Souza, Itamara F. Leite, Suedina M. L. Silva, Marcus V. L. Fook

**Affiliations:** 1 Graduate Program in Materials Science and Engineering, Federal University of Campina Grande, Campina Grande 58429-900, PB, Brazil; 2 Graduate School of Science and Technology, University of Tsukuba, Tsukuba 305-8577, Japan; baah.damasceno@gmail.com (B.C.D.); azvdo.livia@gmail.com (L.A.S.); 3 Institute of Life and Environmental Sciences, University of Tsukuba, Tsukuba 305-8577, Japan; 4 The Alliance for Research on the Mediterranean and North Africa (ARENA), University of Tsukuba, Tsukuba 305-8577, Japan; nakajima.m.fu@u.tsukuba.ac.jp; 5 Materials Engineering Department, Federal University of Paraíba, João Pessoa 58051-900, PB, Brazil; itamara.leite@pq.cnpq.br; 6 Materials Engineering Department, Federal University of Campina Grande, Campina Grande 58429-140, PB, Brazil; suedina.maria@professor.ufcg.edu.br (S.M.L.S.); marcus.fook@ufcg.edu.br (M.V.L.F.)

**Keywords:** high pressure, Ultrasonicator, nanosystem, O/W

## Abstract

The global demand for food has been increasing, presenting new challenges in meeting this demand. To address this growing need, the use of coating technology through nanoemulsions shows great potential. The use of thyme essential oil stabilized by chitosan nanoparticles offers a promising and sustainable approach for the development of edible coatings. Chitosan was extracted from shrimp shell waste and used to produce nanoparticles via the ionotropic gelation method, using sodium tripolyphosphate (TPP) as a crosslinking agent. To prepare the nanoemulsions, thyme essential oil was used as the dispersed phase, combined with an aqueous phase containing chitosan nanoparticles and Tween 80 as the emulsifier. Two techniques were employed to produce nanoemulsions: high-pressure homogenization and ultrasonication. Nanoemulsion formulations with different concentrations were prepared and characterized in terms of droplet size (Z-Average) and stability using dynamic light scattering (DLS). The average droplet sizes obtained were above 100 nanometers for samples produced via high-pressure homogenization and below 100 nanometers for those prepared using ultrasonication. Analysis of variance (ANOVA) confirmed that both the method (*p* = 0.002) and the oil phase concentration (*p* < 0.001) had statistically significant effects on droplet size. Regression analysis showed that oil concentrations below 2.0 g (*w*/*w*) increased droplet size, while concentrations above 4.0 g (*w*/*w*) significantly reduced it (*p* < 0.05). However, physical stability tests conducted at 5 °C for 30 days showed consistent values across both formulations, with only minor fluctuations, suggesting overall good stability.

## 1. Introduction

The consumption of organic foods has been increasing due to the growing global demand, making several factors important, including the expansion of agricultural production [1]. To meet these demands, agricultural producers adopt intensive farming practices, which can lead to increased pesticide use and, consequently, pose risks to human health [2,3]. Food deterioration can result from diseases caused by bacteria and fungi, which function as vectors of Foodborne Diseases. Preventing these contaminants from harming agricultural production is essential for food security. Therefore, effective alternatives are needed to prevent the incidence of pathogens in food [4]. As an alternative for controlling Foodborne Diseases, researchers have been developing food coating technologies that play a key role in preserving fruit quality and extending shelf life. The application of coatings on fruits has become an effective practice to minimize production losses caused by pathogens. Among the most widely used materials for fruit coating, chitosan stands out due to its enormous potential. It is a biopolymer obtained from shrimp shell waste, making its use a sustainable alternative [5,6,7]. The use of chitosan as an edible coating for food preservation has been gaining increasing interest in the academic field due to its functional properties, among them its excellent ability to form transparent films with strong adhesion to the surface of various fruits. As a physical barrier, chitosan helps to delay oxidative processes and moisture loss. This property, combined with its intrinsic antimicrobial activity, makes chitosan one of the most widely used materials in the development of edible coatings [8,9]. In addition, its potential as a polysaccharide to stabilize nanoemulsions highlights chitosan as a promising material with great application potential [10]. Essential oils such as thyme oil have attracted increasing attention due to their antimicrobial and antioxidant activities, which can be enhanced when incorporated into nanoemulsion systems [11]. At the nanometric scale, there is a challenge in developing technological structures that synergistically combine nanomaterials with other substances, such as essential oils. This association at the nanoscale not only amplifies the desired effects but can also enhance the stability, bioavailability, and functional efficacy of the active compounds [12]. High-pressure homogenization and ultrasonication are among the most widely used techniques for nanoemulsion production. However, comparative studies evaluating the influence of these high-energy methods on the physicochemical properties and long-term stability of nanoemulsions stabilized specifically by chitosan nanoparticles and containing thyme essential oil remain limited [13,14,15,16]. In this study, we aim to obtain an oil-in-water (O/W) nanoemulsion of thyme essential oil stabilized by chitosan nanoparticles for potential application as a fruit coating. The following steps will be conducted: analysis of thyme essential oil; production of chitosan nanoparticles using the ionotropic gelation method; preparation of an O/W nanoemulsion; and optimization of the nanoemulsion production process using two distinct methods, Ultrasonicator and High-pressure homogenization. The chitosan nanoparticles and nanoemulsions will be physicochemically characterized in terms of morphology, size, surface charge, suspension homogeneity, and colloidal stability.

## 2. Materials and Methods

### 2.1. Materials

The white thyme essential oil (*Thymus vulgaris* L.) was purchased from the Casa Negri (João Pessoa, Brazil), and stored in amber glass bottles, batch number 0423-002143, for use in the preparation of the nanoemulsion. Chitosan was obtained through the deacetylation process of *Litopenaeus vannamei* shrimp waste, to produce chitosan nanoparticles. Polysorbate derived from plant sources (Polysorbate, from plants), with CAS number 9005-65-6, was purchased from FUJIFILM Wako Pure Chemical Corporation (Osaka, Japan). Soybean oil from the Wako 1st Grade line (CAS 8001-22-7) was also purchased from FUJIFILM Wako Pure Chemical Corporation. Additionally, the reagents glacial acetic acid PA from Química Moderna (João Pessoa, Brazil) and sodium tripolyphosphate (Sigma-Aldrich, St. Louis, MO, USA) were used.

### 2.2. Obtaining Chitosan

The methodology proposed for obtaining chitosan from shrimp waste was based on an adapted method from de Queiroz et al. [17]. The demineralization process was carried out using a 6.7% (*w*/*v*) hydrochloric acid (HCl) solution at a 1:9 (g/mL) ratio, with shrimp shell powder immersed for 3 h at room temperature (25 °C). After this step, the residues were washed with distilled water until reaching a neutral pH between 6 and 7. Subsequently, deproteinization was performed using a 10% (*w*/*v*) sodium hydroxide (NaOH) solution at 80 °C, in a ratio of 1:12.5 (g/mL) of demineralized shells, under mechanical stirring at 350 rpm for 3 h. This process was carried out in two stages. Afterward, the residues were washed with distilled water until reaching a neutral pH between 7 and 8, and then dried in an oven at 90 °C. The deacetylation step was performed using the chitin obtained, with a 50% (*w*/*v*) sodium hydroxide (NaOH) solution at a ratio of 1:36.9 (g/mL) of chitin. The solution was then heated under mechanical stirring at 250 rpm for 6 h at 120 °C to obtain chitosan. After this step, the material was washed with distilled water until reaching a neutral pH between 7 and 8, then dried in an oven at 90 °C for 2 h.

### 2.3. Preparation of Chitosan Nanoparticles

The nanoparticles were obtained using the methodology adapted from Lêoncio et al. [18]. Initially, chitosan was dissolved in a 1% (*w*/*v*) glacial acetic acid solution, in a proportion of 0.0025 g/mL, under magnetic stirring for 1 h at room temperature (25 °C). The chitosan solution was then subjected to an Ultrasonicator to reduce the particle size. A sodium tripolyphosphate (TPP) solution at 1% (*w*/*v*) was prepared under magnetic stirring for 30 min at room temperature and added to the chitosan solution in a 3:1 Chitosan:TPP ratio. The pH value was adjusted to 4.5. After pH adjustment, the solution was stirred for 30 min and subsequently centrifuged at 3000–4500 rpm, at 5 °C for 10 min, to obtain the nanoparticles.

### 2.4. Preparation of Oil-in-Water Emulsion

The nanoemulsion of thyme essential oil will be prepared using 2% (*w*/*w*) of thyme essential oil (EO) and 2% (*w*/*w*) of soybean oil (SO) as the dispersed phase (DP). In the continuous phase (CP), the surfactant Tween 80 (TW) was used at a concentration of 1% (*w*/*w*), along with 75% (*w*/*w*) distilled water (DW) and 20% (*w*/*w*) of the previously obtained chitosan nanoparticle solution (CNP). The mixture was first homogenized using a high-speed homogenizer at a moderate speed (10,000 rpm) for 5 min. Two different methods were used for the nanoemulsion formation: high-pressure homogenization at 100 MPa for one cycle, and ultrasonication for 5 min at 15 watts of power. Different concentrations were obtained to compare the nanoemulsion preparation methodologies [19]. In Table 1, it shows the experimental variables and factor coding used in the study and Table 2, different concentrations were used to compare the nanoemulsion preparation methods, namely High-Pressure homogenization and Ultrasonicator. A Central Composite Design (CCD) was employed to define experimental parameters using Minitab 17 software. These experiments were conducted with the average droplet size as the response variable.

### 2.5. Gas Chromatography–Mass Spectrometry (GC-MS)

To identify the secondary metabolites in thyme essential oil, Gas Chromatography–Mass Spectrometry (GC-MS) was used, employing a Clarus 680 Gas Chromatograph and a Clarus SQ 8 S Mass Spectrometer (PerkinElmer, Waltham, MA, USA). The chromatographic column was Elite-5MS (30 m × 0.25 mm × 0.25 µm). The analyses were carried out using methanol as the solvent at a concentration of 1000 ppm, with a 1 µL injection volume and an injector temperature of 250 °C. The oven temperature program started at 35 °C (4 min), increased to 110 °C at 3 °C/min (held for 3 min), then to 140 °C at 4 °C/min (held for 4 min), with a total runtime of 43.5 min. Detection parameters included a scan range of 10–650 *m*/*z*, ion source temperature of 220 °C, and transfer line temperature of 250 °C. Compound identification was performed by comparing retention times and mass spectra with the NIST spectral library.

### 2.6. Fourier Transform Infrared Spectroscopy (FTIR)

Absorption spectrum was obtained using the FTIR instrument (Spectrum 400, PerkinElmer, Waltham, MA, USA) to determine the degree of deacetylation of chitosan, to analyze the pure essential oil, and to assess possible interactions between chitosan and the essential oil after the production of the nanosystem. The analysis was performed using a Fourier Transform Infrared spectrophotometer equipped with an Attenuated Total Reflectance (ATR) mode, in the range of 4000 to 650 cm^−1^, with a resolution of 4 cm^−1^ and 32 scans.

### 2.7. Degree of Deacetylation

The degree of deacetylation is an essential characteristic used as a parameter for determining the properties of chitosan. To evaluate the degree of deacetylation, the potentiometric titration method was employed. In this procedure, a chitosan solution (1 g/L) was prepared in 0.02 mol/L HCl and subsequently titrated with 0.2 mol/L NaOH, while the pH was continuously monitored. The resulting pH curve revealed two inflection points, and the difference in NaOH volume between these points corresponded to the amount of acid consumed during the protonation of the amino groups of chitosan, thereby allowing the calculation of its degree of deacetylation through the applied Equation (1) [20].

(1) $$DD\;(\%)=\frac{V_{NaOH}\;(L)\times C_{NaOH}\;(\mathrm{mol}/L^{-1})\times M_{glucosamine}\;(g/{\mathrm{mol}}^{-1})\;}{m_{sample\;(g)}}\;\times 100$$

### 2.8. Dynamic Light Scattering (DLS) and Zeta Potential Analysis

To measure the size and stability of the nanoparticles, dynamic light scattering (DLS) analysis was performed using a Malvern Panalytical model, with the sample solution at room temperature. The zeta potential of the samples was determined by electrophoretic light scattering (ELS) measurements using the same instrument. Each sample was placed in a folded capillary cell and loaded in the instrument. The ζ-potential of samples were automatically measured using 10–100 runs per analysis after they were equilibrated for 120 s inside the instrument at 25 °C.

### 2.9. pH Determination

To determine the pH, measurements were performed using a pH meter (Quimis, model TEC-2, Brazil) which had been previously calibrated with buffer solutions of pH 4.0 and 7.0. The pH of the sample was measured, and all analyses were performed in triplicate.

## 3. Results and Discussion

### 3.1. Chemical Composition Identified by GC–MS 

**Figure 1:**
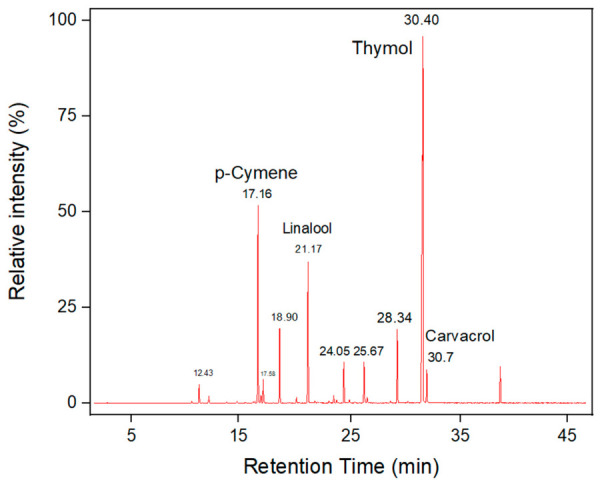
Chromatogram of Thyme Essential Oil.

We can observe the chromatographic results in Figure 1, with the largest peak corresponding to thymol, as expected from the literature. Thymol was found at 33%, followed by p-cymene at 18%, and linalool at 12%, among other compounds and their respective retention times. Based on these results, it can be affirmed that the thyme essential oil contains the identified compounds consistent with the literature, characterizing *T. vulgaris* L. essential oil as having antimicrobial and antioxidant potential [21].

Different results have been reported in the literature; for example, found a higher concentration of thymol (22%) in the analyzed essential oil, whereas in this study it was 33%. Similarly, p-cymene was the second most abundant compound with approximately 21% in the article and 18% in this analysis. Other compounds showed differences; for instance, reported carvacrol as the next most abundant compound at 13%, whereas in this study it was linalool at 12%, which was only 5.58% in the article. Higher values of thymol (up to 47%) and approximate values of linalool have also been reported [22].

The 33% thymol content found here is consistent with literature values, but it is important to highlight that different environmental conditions influence the plant’s defense mechanisms, leading to varying concentrations of components such as carvacrol and p-cymene [22]. Thymol was effective against *Aspergillus niger* in post-harvest fruits such as blueberries. When combined with chitosan, its efficacy against bacteria is enhanced; for example, it showed effectiveness in inhibiting *Escherichia coli* [23,24]. In general, thymol associated with p-cymene has demonstrated significant effects against *Klebsiella oxytoca*, *Bacillus cereus*, and *Staphylococcus aureus* [25]. The inhibitory capacity of thyme essential oil was observed against pathogens such as *Bacillus cereus*, *Listeria monocytogenes*, *Enterococcus faecalis*, *Aspergillus*, *Penicillium*, *Mucor*, and others [26].

In total, 23 compounds are present in the essential oil as shown in Table 3. The chemical composition of essential oils is highly variable and depends on several environmental, biological, agronomic, and meteorological factors, as plants produce these secondary metabolites as a means of adapting to their surroundings. In the case of *T. vulgaris*, the profile of volatile compounds such as thymol, carvacrol, p-cymene, and γ-terpinene can vary significantly.

### 3.2. FTIR Spectral Analysis

**Figure 2:**
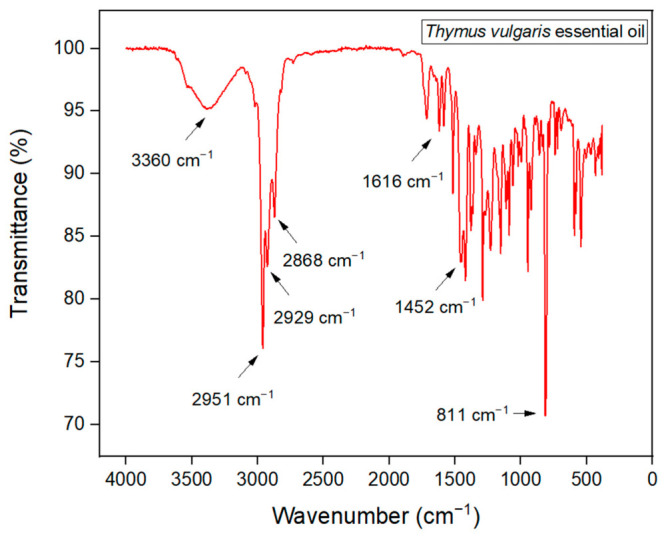
FTIR Spectrum of Thyme Essential Oil.

The thyme essential oil was analyzed by Fourier Transform Infrared Spectroscopy (FTIR) using ATR mode to confirm the presence of functional groups, as shown in Figure 2. Combining the results from Gas Chromatography and FTIR helps to corroborate and better interpret the data.

In the obtained spectrum, a broad band around 3360 cm^−1^ is observed, attributed to the O–H stretching of phenols, indicating the presence of phenolic compounds such as thymol and carvacrol, known for their antioxidant and antimicrobial properties associated with their aromatic rings, similar to results found in the same region [27].

In the region between 2951 cm^−1^ and 2929 cm^−1^, characteristic bands of C–H stretching from methyl and methylene groups, respectively, and their aliphatic chains appear, suggesting the presence of hydrocarbon chains typical of terpenes. Additionally, a peak at 2868 cm^−1^ corresponds to the symmetric vibration of these groups, though with lower intensity, while a stronger intensity is found at 1452 cm^−1^. In this case, the presence of p-cymene can be verified, which also corroborates the results found in the literature [22].

A peak around 1616 cm^−1^ can be related to stretching vibrations of C=C double bonds, characteristic of aromatic structures and the benzene ring present in compounds such as thymol and linalool. The region between 1500 cm^−1^ and 1000 cm^−1^ shows multiple intense bands associated with C–O stretching vibrations, compatible with the presence of alcohols and ethers, and characteristic of C–OH bonds in phenols. Below 1000 cm^−1^, several peaks are observed due to the chemical complexity of the oil and may be associated with the presence of specific terpenes structures. At the 811 cm^−1^ peak, an out-of-plane bending of hydrogen atoms attached to the aromatic ring can be observed. This vibration occurs when hydrogen atoms move perpendicular to the plane of the benzene ring. Overall, the FTIR spectrum confirms the presence of functional groups consistent with the main constituents of thyme essential oil [27]. Variations in FTIR spectra are normal due to different secondary metabolites in the plant, such as a reduction in the peak around 860 cm^−1^ caused by a low concentration of carvacrol [28].

**Figure 3:**
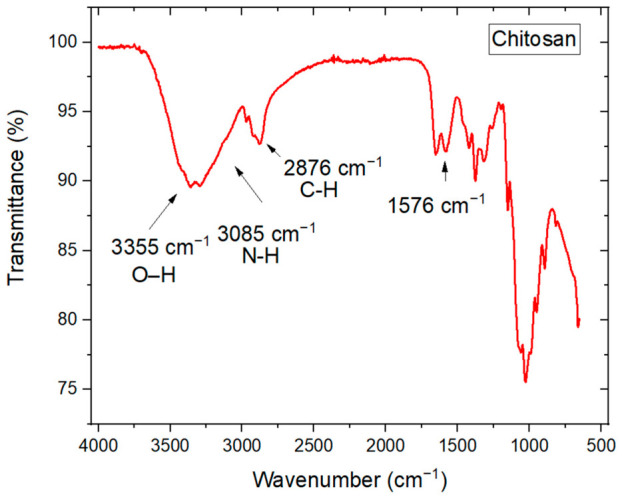
FTIR Transmittance Spectrum of Chitosan Obtained.

Figure 3 shows the FTIR spectrum performed to confirm the presence of characteristic functional groups of chitosan obtained from *Litopenaeus vannamei* shell waste. In the spectrum, a broad band around 3600 cm^−1^ to 3000 cm^−1^ can be highlighted, which is associated with the stretching vibrations of hydroxyl O–H and amine N–H bonds. These results are consistent with those found in the literature. In this region, a specific peak at 2876 cm^−1^ corresponds to the C–H stretching of aliphatic groups present in the polymer structure, also reported in the same study [29]. At 1685 cm^−1^, a peak is attributed to the C=O stretching vibration of Amide I, which is similarly observed at 1665 cm^−1^ in other studies [30]. Additionally, peaks at 1636 cm^−1^ and 1576 cm^−1^ correspond respectively to Amide II (N–H bonds) and Amide III (C–N bonds), with values matching those found in the literature, confirming the deacetylation process [31].

Based on these results, it can be concluded that chitosan was successfully obtained. The presence and position of these bands confirm the identity of the material as chitosan and demonstrate the efficiency of the extraction and deacetylation process. These findings are consistent with data reported in the literature [32].

### 3.3. Degree of Deacetylation of Chitosan

**Figure 4:**
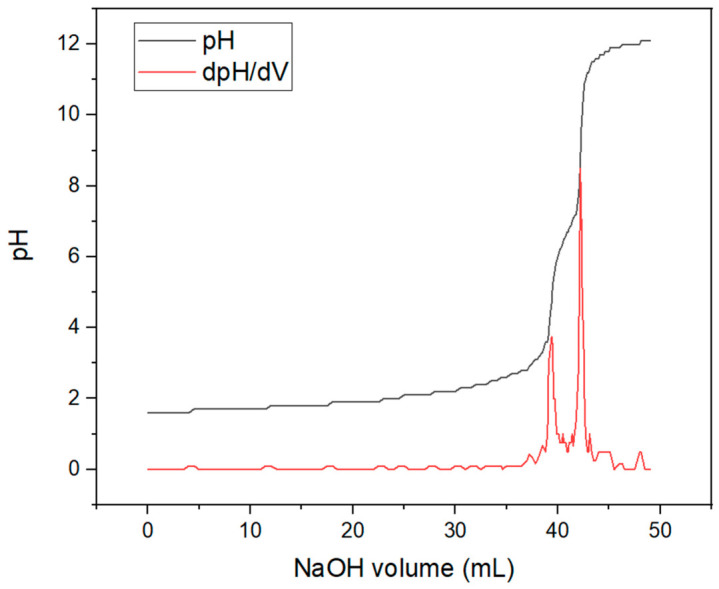
pH Method Curve for Determining the Degree of Deacetylation.

Figure 4 shows the potentiometric titration curve and its derivative, used for determining the degree of deacetylation of chitosan. The application of Equation (1) allowed obtaining a degree of deacetylation of 83.15%, a value consistent with data reported in the literature, which confirms the successful production of chitosan [33]. A comparison was made with the method described in the referenced study, where similar values were found [34]. The titration of sodium hydroxide in the chitosan solution produced a curve along with its pH derivative, and the volume of sodium hydroxide at which an inflection point could be identified.

### 3.4. Chitosan Nanoparticles

Chitosan nanoparticles were obtained through the ionotropic gelation technique, a widely used method due to its simplicity, low toxicity, and ability to produce stable particles in an aqueous medium. This process is based on the formation of bonds between the amine groups of chitosan and crosslinking agents such as sodium tripolyphosphate (TPP). The interaction between these opposite charges leads to the spontaneous formation of a three-dimensional network, resulting in the precipitation of nanoparticles. In the present study, the produced nanoparticles had an average hydrodynamic diameter of 280 nanometers, as determined through dynamic light scattering (DLS) analysis. Similar results were found, although using different concentrations in the methodology in Hejjaji et al. [35].

Chitosan, with a lower degree of deacetylation, however, will result in smaller particle sizes [36]. The particle size can be influenced by several factors, including the ratio of chitosan to TPP, the concentration of the reagents, the pH of the solution, and the stirring time during the crosslinking process. Studies that optimized the production process achieved results below 200 nanometers [37]. The obtained particles showed good uniformity, without signs of visible agglomeration, suggesting satisfactory colloidal stability. The production of nanoparticles with an average size in the range of 280 nanometers indicates the efficiency of the employed methodology. The Zeta potential was +38 mV, above the stability range found in the literature. Chitosan nanoparticles obtained by ionic gelation showed a zeta potential of +38 mV, a value that indicates high colloidal stability of the system. Values above the stability range were also found in Khoerunnisa et al. [37]. 

Zeta potential is a measure of the electrostatic repulsion between particles in suspension; values above +30 mV or below –30 mV is generally considered indicative of stable dispersions [38]. In the case of chitosan nanoparticles, the positive value reflects the presence of protonated amine groups on the surface of the nanoparticles, which favors repulsion between particles and prevents aggregation [39]. Furthermore, this positive charge can facilitate interaction with negatively charged surfaces, which is especially relevant for applications in active coatings with antimicrobial properties [40].

### 3.5. Nanoemulsions by High Pressure Homogenization

The nanoemulsions obtained by high-pressure homogenization were prepared using 2% (*w*/*w*) of the dispersed phase. They presented a whitish appearance, which is common in nanoemulsions due to their physical properties. It is worth noting that this appearance is completely different from that of conventional microscale emulsions. This occurs due to light scattering when it is refracted multiple times through the droplets, which gives nanoemulsions their characteristic appearance [41]. The combined use of the two oils enabled the formation of a homogeneous and stable nanoemulsion due to the differences in the densities of the respective oils. This characteristic is important in the preparation of the dispersed phase, and such factors, such as density differences, must be taken into consideration, as they help prevent the degradation of compounds present in the essential oil [42].

**Figure 5:**
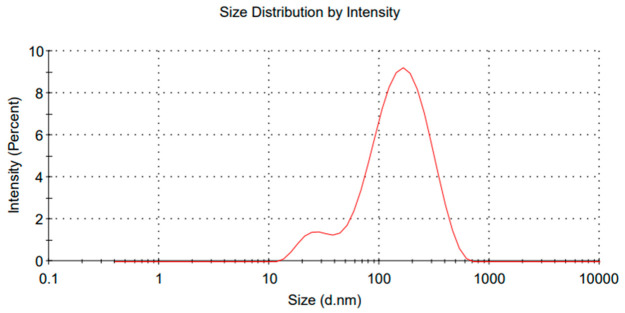
High-Pressure Nanoemulsion O/W (104 nm Average Size).

In Figure 5, the average size of the nanoemulsion obtained by high-pressure homogenization was approximately 104 nanometers. The Intensity (%) represents the relative contribution of droplets of different sizes to the total scattered light signal detected during the measurements. This representation emphasizes larger droplets due to their stronger light scattering behavior. A similar result was found in the literature with 99 nanometers using thyme oil, although that study employed a traditional method with five homogenization cycles instead of just one [43]. It is common to find droplet sizes ranging from 100 to 140 nanometers when using high-pressure methods, even with different oils or polysaccharides used as stabilizers [44].

The zeta potential was +1 mV, indicating that the nanoemulsions were unstable. According to the literature, a zeta potential below ±30 mV suggests low stability of the nanoemulsion against flocculation or coalescence [45]. A value within the range of –30 mV to +30 mV indicates that the system exhibits low electrostatic stability. Zeta potential is a key parameter for assessing the colloidal stability of emulsions and nanoemulsions, as it is related to the repulsion between charged particles in dispersion. Absolute values lower than ±30 mV generally indicate that the repulsive forces are not sufficient to prevent droplet aggregation and coalescence, which may lead to phase separation over time [46].

**Figure 6:**
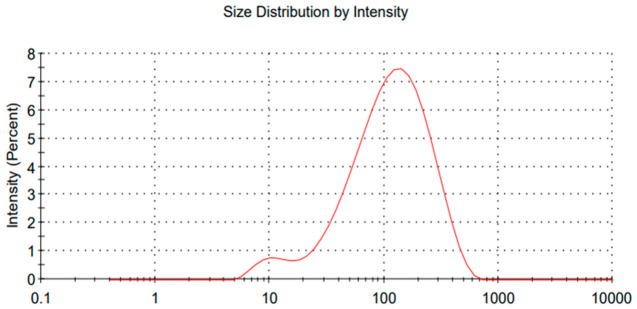
High-Pressure Nanoemulsion O/W after 4 months (133 nm Average Size).

The stability of nanoemulsions was evaluated, and it was observed that after 20 days, the average droplet size was 123 nanometers, while the zeta potential remained stable. This result demonstrated better performance compared to a study that used chitosan and essential oil, where the initial droplet size was 139 nanometers, increasing to 182 nanometers after 21 days. It is important to note that the zeta potential in that study was also within the instability range [47]. The evaluation of nanoemulsion stability over time is essential to ensure its effectiveness in practical applications. After 20 days of storage, the average droplet size remained stable, indicating good resistance to coalescence and flocculation. The maintenance of the mean droplet size suggests that additional stabilization mechanisms may be acting within the system [48]. These mechanisms can be attributed to the presence of polycationic chitosan, which can adsorb at the oil–water interface, forming a dense protective layer around the droplets. This layer acts as a steric barrier, preventing close contact between droplets and thereby reducing the risk of coalescence or flocculation. Additionally, the increased viscosity of the continuous phase may contribute to this effect. Such steric stabilization compensates for the insufficient electrostatic repulsion to ensure long-term stability [49]. Monitoring the size of nanoemulsions over time is one of the main indicators of their physicochemical stability [49]. After 2 months of storage, it was observed that the average droplet size remained around 130 nanometers, indicating that the system exhibited good colloidal stability during this period. The observed stability can be attributed to the efficiency of the High-pressure homogenization process used in the production of nanoemulsion. Similar results were obtained using lectin as a nanoemulsion stabilizer; however, using canola oil, the nanoemulsions were not stable [50]. After 4 months, the size of the nanoemulsions can be seen in Figure 6, averaging 133 nanometers.

After 4 months of storage, the nanoemulsion remained stable from a physicochemical point of view, even showing variations in the average droplet size compared to the first two months. Similar results with chitosan were found, demonstrating its potential for stability in nanoemulsions. This variation in the initial peak being bimodal initially reflects the presence of unstable smaller droplets. However, after 4 months, a monomodal distribution can be observed, indicating controlled aggregation, causing the smaller droplets to disappear and bringing uniformity to the nanoemulsion [51].

### 3.6. Nanoemulsions by Ultrasonicator

**Figure 7:**
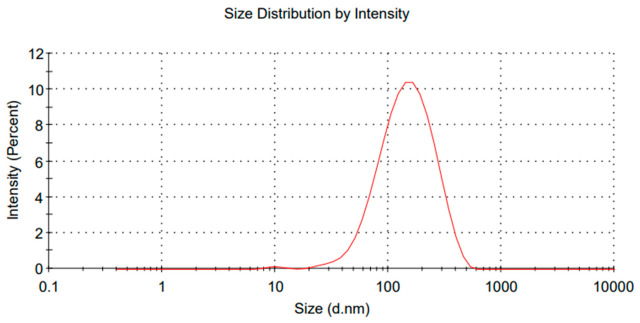
Ultrasonicator Nanoemulsion O/W (77 nm Average Size).

The nanoemulsions obtained by Ultrasonicator were prepared using 2% (*w*/*w*) of the dispersed phase. They showed the common appearance expected for nanoemulsions. The droplet size result obtained for the nanoemulsion using the Ultrasonicator method was, on average 77 nanometers, indicating the formation of highly dispersed and stable nanometric droplets. as shown in Figure 7.

This size is significant because smaller particles generally provide greater stability for emulsion due to reduced coalescence. Similar results can be found using the same methodology, where the nanoemulsions reached 80 nanometers [15]. Ultrasonication is a widely used technique for obtaining nanoemulsions, as it promotes the efficient disruption of the oil and aqueous phases through acoustic cavitation, resulting in droplets with narrow distribution and reduced sizes. A nanoemulsion size below 80 nanometers indicates that the parameters of power, frequency, and time were appropriate to achieve a dispersion with optimal results, also favoring the increase in the droplets’ surface area, which enhances the functional properties of the nanoemulsion. The physical stability also tends to be higher in nanoemulsions, reducing the tendency for flocculation and phase separation over time [52]. The production of such small nanoemulsions by ultrasonication, using chitosan to stabilize the nanoemulsion, reinforces the potential of this methodology as a sustainable and effective alternative for the incorporation of essential oils, since the use of chitosan allowed results below 100 nanometers, making this methodology more efficient [53].

The zeta potential value of the O/W nanoemulsion obtained by Ultrasonicator was approximately +4 mV, which indicates low electrostatic stability of the colloidal system. In general, zeta potential is a parameter used to assess the physicochemical stability of colloidal dispersions, such as nanoemulsions. Absolute zeta potential values above ±30 mV usually indicate systems with stable charges due to strong electrostatic repulsion between the droplets. The use of chitosan has yielded values below the desired range when applied in nanoemulsions [54]. The presence of an average size of 77 nanometers suggests that the nanoemulsion exhibits good initial dispersion and that the mechanical action of ultrasonication was effective in reducing droplet size. However, it is important to highlight that nanoemulsions with low zeta potential lack an electrostatic barrier, which may lead to instability. In this case, stability can be maintained through a steric barrier and increased viscosity, preventing close contact between droplets and thus reducing the risk of coalescence or flocculation [48]. An average diameter of 78 nanometers after 20 days of storage indicates satisfactory stability of the nanostructured system over time, with minimal size variation compared to the previously mentioned initial value of 77 nanometers. The fact that the emulsion maintained a nanometric average size close to the original value, even after 20 days, suggests that there was no significant droplet growth due to coalescence or flocculation, as would be expected in unstable colloidal systems.

This demonstrates that ultrasonication was effective in producing an emulsion with good dispersion and that the chitosan nanoparticles used to stabilize the nanoemulsion played a role in preventing its instability. When using other parameters to stabilize thyme essential oil, different results can be observed compared to those obtained with chitosan. This should be taken into consideration, because despite similar droplet sizes, the stability provided by chitosan leads to better overall emulsion stability [55].

**Figure 8:**
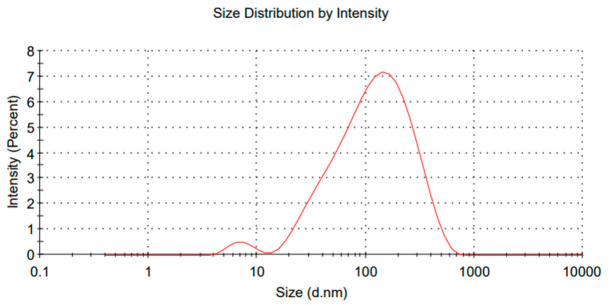
Ultrasonicator Nanoemulsion O/W (81 nm Average Size).

Thyme essential oil nanoemulsions with droplet sizes below 100 nm are desirable for food industry applications, as they provide greater transparency and also enhance biological activity against microorganisms [56]. The maintenance of the nanoscale over time is one of the main success criteria in formulations intended for technological applications. It is important to highlight that size stability over time also depends on factors such as proper storage. The variation from the initial value was minimal, indicating that no significant coalescence occurred. This result reinforces the efficiency of the ultrasonication process and the effectiveness of the formulation in maintaining the nanometric dispersion of the droplets. In another study, chitosan combined with a different essential oil using alternative methods showed signs of Ostwald ripening after 60 days [57]. In contrast, after 4 months, the average droplet size of the nanoemulsions in this study was 81 nanometers, as shown in Figure 8.

Compared to the previous data, 77 nanometers (initial), 78 nanometers (20 days), and 80 nanometers (2 months), it can be concluded that there was only a very slight variation in the average droplet diameter, with an increase of just 4 nanometers over 120 days. This behavior indicates significant colloidal stability. The absence of visible droplet growth suggests that processes such as coalescence, flocculation, or Ostwald ripening were minimized or did not occur. Different results were observed by Phyo et al. [58].

The observed stability can be attributed to both the efficiency of the ultrasonication process, which promoted a homogeneous size distribution during preparation, and to the action of the chitosan nanoparticles used, which provided adequate protection at the oil-water interface. Even with a relatively low zeta potential, as discussed previously, the emulsion remained stable, indicating that steric stabilization mechanisms may be playing a predominant role.

This maintenance of the nanoscale for 4 months is highly relevant, as nanoemulsions with droplet sizes below 100 nanometers ensure high surface area, better dispersion of bioactive compounds, and, in the case of edible coatings, offer strong potential for application, for example, good adhesion and transparency, which are essential factors for both sensory and functional acceptance in food products. This result aligns with previous findings in the literature, where nanoemulsions composed of chitosan and thyme essential oil, though aimed at non-food applications, showed no significant changes after 90 days. A noteworthy difference is that the referenced samples were stored at room temperature, whereas in our case, the samples were refrigerated [59].

### 3.7. Comparison of Nanoemulsion Preparation Methods

Different values were obtained for each parameter, and the results show varying central point values for different methodologies, High-pressure homogenization and Ultrasonicator, as shown in Table 4. The results obtained using the High-pressure method reached central point values between 105 and 108 nanometers in experiments 1, 2, and 5. On the other hand, the Ultrasonicator method achieved values between 82 and 87 nanometers in experiments 9, 12, and 13. It can be concluded that the Ultrasonicator method produces smaller values compared to the High-pressure method.

This can be explained by the mechanisms involved in each of the applied methodologies. In the High-pressure method, pressure is used to generate shear forces that promote orthokinetic collisions, which differ from the mechanism used in ultrasonication. Ultrasonication relies on acoustic cavitation, and it also uses shear forces to reduce droplet size. However, a key difference is that it utilizes bubble implosions to generate localized high energy, which contributes to the formation of nanoemulsions [60].

In the experiments conducted using High-pressure Homogenization, the results obtained from experiments 1, 2, and 5 indicated average droplet sizes of 105, 106, and 108 nanometers, respectively. All three experiments were carried out under the same conditions with an oil phase concentration of 4.0% (*w*/*w*), which allows us to state that these formulations are close to the experimental central point of the adopted design. These values reveal good reproducibility of the process, as there is a very small variation of only 3 nanometers between the results, indicating consistency in the parameters used and effective control of the conditions during the preparation experiments. This uniformity is a positive indicator of the system, especially with a view toward industrial processes where repeatability is essential.

The average droplet size obtained, ranging from 105 to 108 nanometers, falls within the nanoscale range and is functional for oil-in-water emulsions. Although these values are higher than those observed with ultrasonication, they are still considered satisfactory. Furthermore, the size obtained by high-pressure homogenization depends on the applied pressure. Since the system remained within the nanoemulsion range, it can be concluded that the applied pressure was effective in producing nanodroplets.

Comparatively, although ultrasonication generated smaller particles, high-pressure homogenization offers important advantages such as greater industrial scalability, lower risk of thermal degradation, and the possibility of continuous processing in large volumes. This makes the results obtained at the central point, ranging from 105 to 108 nanometers, quite relevant. The Sonicator method yielded values between 82 and 87 nanometers in experiments 9, 12, and 13. We can state that the Ultrasonicator method was able to produce smaller droplet sizes compared to the High-pressure method. The average droplet size of the nanoemulsions obtained by ultrasonication demonstrates the technique’s ability to produce emulsions with good dispersion and homogeneous distribution, key characteristics for applications that demand physicochemical stability and high functional performance. In the experiments carried out using the Ultrasonicator method, a variation in droplet size was observed, ranging from 82 to 87 nanometers. However, when focusing on the central points of the experimental design, specifically experiments 12 and 13, both were conducted using 4.0% (*w*/*w*) aqueous phase.

These results indicate that, under optimal formulation conditions, the ultrasonication process is highly effective in forming particles with well-distributed sizes within the desired nanometric range. In contrast, experiment 8, which used a different proportion of 2.0% (*w*/*w*) oil phase, resulted in a considerably larger droplet size of 130 nanometers. This demonstrates that the performance of the Ultrasonicator is directly related to the oil phase proportion, and values below the central point may compromise the efficiency of nanoemulsion formation possibly due to lower viscosity or reduced droplet density.

The consistency of the results in experiments 12 and 13 confirms that when the formulation parameters are appropriate, the Ultrasonicator method shows excellent performance in producing nanoemulsions with a low average droplet size, high dispersibility, and potential for greater physicochemical stability, even in systems with relatively low zeta potential. When compared with the data obtained using the High-pressure method, which showed central values between 105 and 108 nanometers, it becomes evident that the Ultrasonicator method is more efficient in reducing particle size. This difference can be attributed to the nature of the acoustic cavitation mechanism promoted by the Ultrasonicator, which results in a more intense and localized droplet disruption.

It was observed that the higher the amount of oil used in the oil phase when using High-pressure homogenization, the smaller the droplet size. Using 1% (*w*/*w*) of each oil resulted in values above 120 nanometers. A significantly lower droplet size was obtained when using 3% (*w*/*w*) of each oil, with droplets below 80 nanometers. Some hypotheses may explain this phenomenon, such as the reduction in interfacial tension, which facilitates emulsification and stabilizes smaller droplets, as well as differences in phase viscosity, which improves the system’s stability [61].

The observed behavior, in which higher oil concentrations result in smaller droplets, may seem counterintuitive but can be justified by the physicochemical mechanisms governing nanoemulsion formation. In the experiments, the formulation containing 1% (*w*/*w*) of each oil led to droplet diameters exceeding 120 nanometers. In contrast, when using 3% (*w*/*w*) of each oil, droplet sizes decreased significantly, resulting in droplets smaller than 80 nanometers. With more oil available, the oil–water interface is better utilized, favoring the formation of a broader and more stable interfacial area. This allows the surfactant to act more effectively, reducing the energy required for droplet dispersion [61]. Furthermore, the viscosity of the dispersed phase tends to increase with the addition of more oil. A slightly higher viscosity can help delay coalescence and flocculation during emulsion formation, stabilizing smaller droplets immediately after their formation. This optimal viscosity helps to dampen collisions between droplets, promoting system stability and reducing particle growth over time [62].

Another important aspect is that higher oil concentrations may lead to a more effective distribution of mechanical energy during the emulsification process. At very low oil concentrations, the total interfacial area is limited, which may not fully optimize the action of the emulsifier. In contrast, at higher concentrations, the increased interfacial area can promote better organization of surfactant molecules, enhancing the system’s efficiency in preventing coalescence and resulting in smaller droplet sizes [63].

The axial points also support the idea that greater variation in the oil phase leads to greater differences in the results, as observed in the values of 70 and 130 nanometers, which represent the lowest and highest droplet sizes, respectively. This highlights the correlation that the amount of oil used has a more significant impact than the method itself. The axial points of the experimental design, those that go beyond the conventional levels of factor variation, offer a broad view of the system’s behavior under extreme conditions. In the present study, they confirmed a trend that the greater the variation in the oil phase, that is, in the dispersed phase, the greater the impact on the particle size. This is evident in the experiments using 1.7% (*w*/*w*) and 6.8% (*w*/*w*) of the oil phase, which resulted in the largest (130 nm) and the smallest (70 nm) particle sizes observed in the entire experimental set, respectively [64].

For the treatment of the data obtained from the CCD, a factor analysis was performed, followed by an analysis of variance (ANOVA using Minitab, version 17), as shown in Table 5, in order to verify whether the results present statistical significance.

The ANOVA showed that both the methods and the concentration of the disperse phase significantly influenced the mean droplet size in the developed nanoemulsions. The method factor presented *p* = 0.002 (F = 21.12), indicating a statistically significant difference between the high-pressure and Ultrasonicator methods, confirming the direct impact of the technique on droplet size reduction.

Even more noticeable was the effect of the oil phase concentration (*p* = 0.000; F = 40.97), evidencing it as the main factor responsible for the observed variation, consistent with the experimental data showing that higher oil concentrations favored the formation of smaller particles. The error term, with DF = 8 and Adj MS = 29.83, was relatively low, reinforcing the reliability of the model. Overall, the ANOVA results demonstrate that both factors significantly affect particle size, with the oil phase concentration having the greatest impact.

Table 6 presents the coefficients of the multiple linear regression model fitted to explain the mean droplet size as a function of the High-pressure and Ultrasonicator methods and the concentration of the disperse phase used in the nanoemulsion formulations. The model is statistically significant, allowing interpretation of the individual effects of each variable. The intercept coefficient (99.97) represents the estimated mean droplet size under the reference conditions. The method has a negative coefficient (−9.17, *p* = 0.002), indicating that switching to the Ultrasonicator reduces the droplet size by 9.17 nm on average, highlighting its efficiency. Regarding the oil phase, low concentrations (1.7% and 2.0% *w*/*w*) show positive coefficients (20.87, *p* = 0.003; 37.20, *p* < 0.001), indicating larger droplet sizes due to lower emulsification efficiency, while higher concentrations (4.0% and 6.0% *w*/*w*) present negative coefficients (−6.13, *p* = 0.038; −12.80, *p* = 0.015), promoting smaller droplets. These results confirm the dominant effect of the oil phase on droplet size, in agreement with the ANOVA findings. Furthermore, all VIF values are below 2, indicating no concerning multicollinearity among predictors and ensuring the model’s statistical stability, whereas values above 5 would suggest strong correlation between factors [65]. Additionally, Table 7 presents the R^2^ model, which complements the results demonstrated in Table 6 regarding the regression analysis.

(2) $$d_{av}=99.97-9.17\times \mathrm{Sonicator}\;+9.17\times \mathrm{High}\;\mathrm{pressure}+20.87\times {OP}_{1.7}+37.20\times {OP}_{2.0\;}-6.13\times {OP}_{4.0}\;-12.80\times {OP}_{6.0}-39.13\times {OP}_{6.8}$$

The presented model, shown by Equation (2), demonstrates an excellent fit to the experimental data, with R^2^ = 95.50% and adjusted R^2^ = 92.68%, indicating that it explains most of the variability in mean droplet size while maintaining robustness considering the number of variables. The residual standard deviation (S = 5.46) reflects relatively low prediction error. The coefficients reveal that Ultrasonication reduces droplet size through cavitation, whereas High-pressure homogenization increases it. The effect of disperse phase concentration is nonlinear: low concentrations (1.7% and 2.0% *w*/*w*) increase droplet size, while higher concentrations (4.0%, 6.0%, 6.8% *w*/*w*) reduce it, with the strongest reduction at 6.8% suggesting a critical point for optimal stability and minimal droplet size. The opposing yet similarly sized effects of the Ultrasonication and High-pressure methods highlight the need to balance these processes, and overall, the results indicate that careful adjustment of emulsification method and oil phase concentration allows precise control and optimization of nanoemulsion droplet size.

Table 8 presents the results of the Tukey test comparing the effects of the two methods used to produce nanoemulsions. The High-pressure method resulted in a significantly larger mean particle size (109.13 nm) compared to Ultrasonication (90.80 nm). The statistical difference between groups is indicated by different letters (^a^ and ^b^), confirming that the methods have distinct effects on droplet size. These results are consistent with the regression model in Equation (1), where Ultrasonication reduces particle size while High-pressure homogenization increases it.

Table 9 shows the results of the Tukey test comparing the effects of different oil phase concentrations on mean particle size. Means followed by different letters indicate statistically significant differences (*p* < 0.05), while those sharing the same letter are not significantly different. Low oil concentrations (1.7% and 2.0% *w*/*w*) formed a group with the largest particle sizes (120.83–137.17 nm, letter “A”), indicating that lower concentrations favor larger droplets. Intermediate concentrations (4.0% and 6.0% *w*/*w*) resulted in smaller particle sizes (93.83 and 87.17 nm, letters “B” and “B C”), suggesting improved interaction between oil and nanoparticles, enhancing stability and reducing coalescence. The highest concentration (6.8% *w*/*w*) formed a distinct group with the smallest droplets (60.83 nm, letter “C”), indicating optimal particle formation and dispersion. The gradual transition between groups reveals a nonlinear effect of oil concentration on particle size, highlighting an optimal range for maximizing droplet size reduction.

In Table 10 a multivariate analysis of variance (MANOVA) was performed to simultaneously assess the effects of oil phase concentration and emulsification method on the system’s response variables. The results indicated that both factors had statistically significant effects, confirming their relevant influence on the evaluated emulsion parameters.

The multivariate analysis evaluated the effects of oil phase concentration and method on the response variables using four criteria: Wilks’ Lambda, Lawley-Hotelling, Pillai’s Trace, and Roy’s Largest Root. For the oil phase, all criteria were highly significant (*p* = 0.000), indicating strong effects on the dependent variables. Wilks’ Lambda (0.0465) shows minimal unexplained variance, Lawley-Hotelling (20.48) reflects a strong effect, Pillai’s Trace (0.953) confirms robustness against assumption violations, and Roy’s Largest Root (20.48) highlights the dimension most discriminating between concentrations. For the emulsification method, all criteria were also significant (Wilks’ Lambda = 0.2747, F = 21.12, *p* = 0.002; Lawley-Hotelling = 2.64; Pillai’s Trace = 0.725; Roy’s Largest Root = 2.64), confirming that both High-pressure homogenization and Ultrasonication significantly affect the variables, though with a smaller magnitude than the oil phase. Overall, MANOVA confirms that oil concentration exerts the strongest influence on particle size and properties, while the emulsification method remains an important factor. These results emphasize the need to control both parameters to optimize nanoemulsion characteristics, ensuring droplet size reduction, stability, and efficient coating formation.

### 3.8. Stability over Time

The stability of the nanoemulsions produced by different methods was evaluated at several time points (Days 01, 07, 14, 21, 30), by droplet size measurements. This approach allowed monitoring of particle size evolution about coalescence, sedimentation, or flocculation. The results provide insights into the durability and stability of nanoemulsions produced by Ultrasonication and High-pressure methods, highlighting their efficiency in maintaining small and uniform droplets over time. Periodic monitoring is essential for optimizing production conditions and ensuring stability for practical applications, such as fruit coatings, preserving antimicrobial and conservation properties.

**Figure 9:**
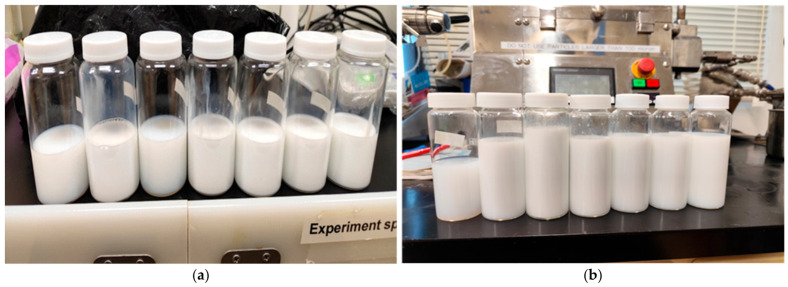
(**a**). Ultrasonication method on Day 01 and (**b**). High-pressure method on Day 01.

**Figure 10:**
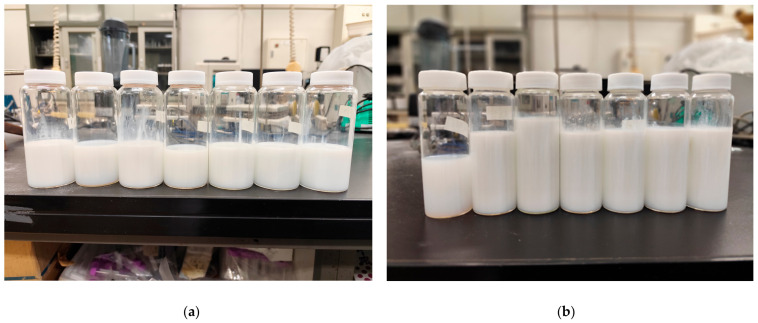
(**a**). Ultrasonication method on Day 30 and (**b**). High-pressure method on Day 30.

After 30 days, the nanoemulsions maintained their visual appearance observed on Day 1, with no phase separation, significant turbidity, or visible precipitation. This visual stability during storage confirms the effectiveness of the formulations in preserving dispersion and the integrity of the nanoemulsion structure. Similar behavior was reported for curcuma-based nanoemulsions after 28 days, although initial average values were lower and Ostwald ripening was observed, unlike in the nanoemulsions produced in this study [66]. Better results with the High-pressure method were achieved, yielding outcomes comparable to Ultrasonication and maintaining stability after 28 days. However, it is important to note that this involved 5 cycles, different from the single cycle used in the emulsions of this study. Additionally, the oil phase composition differed, with soybean oil representing 80% of the disperse phase. These findings suggest that multiple processing cycles and a higher proportion of vegetable oil in the disperse phase can improve the performance of the High-pressure method [67]. As can be observed in Figure 9, the results refer to Day 1 of the nanoemulsions obtained by ultrasonication and high-pressure homogenization methods, while Figure 10 corresponds to Day 30 for the same two methods.

**Figure 11:**
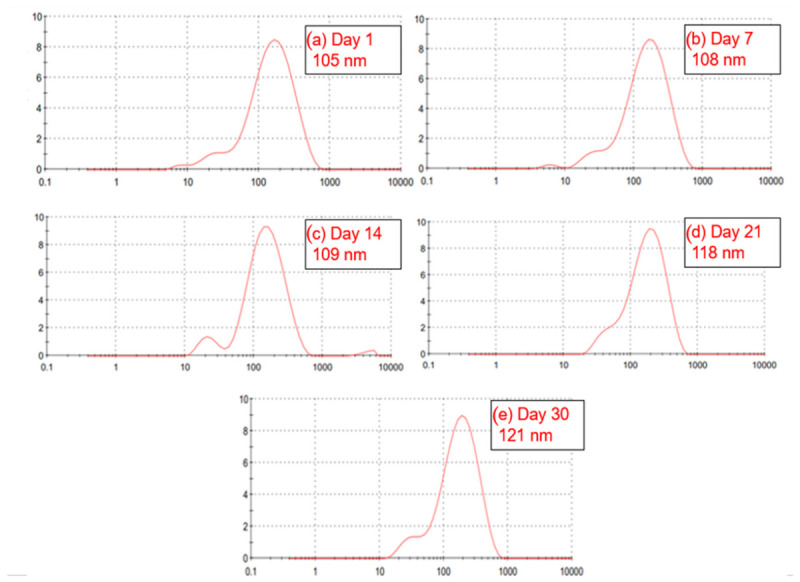
Droplet sizes obtained with High-pressure method over time (**a**) day 1; (**b**) day 7; (**c**) day 14; (**d**) day 21; (**e**) day 30.

In Figure 11 a nanoemulsion prepared by High-pressure homogenization with a 4% (*w*/*w*) oil phase showed a relatively satisfactory stability over the 30 days of monitoring. On the first day, the average particle size was 105 nanometers. A gradual increase was observed over the weeks: 108 nanometers on day 7, 109 nanometers on day 14, and 118 nanometers on day 21. On the thirtieth day, the average size reached 121 nanometers. This variation was progressive but not abrupt, indicating that the emulsion maintained its structure in a stable manner without undergoing severe coalescence or visible phase separation. These results are compatible with those found in [68].

**Figure 12:**
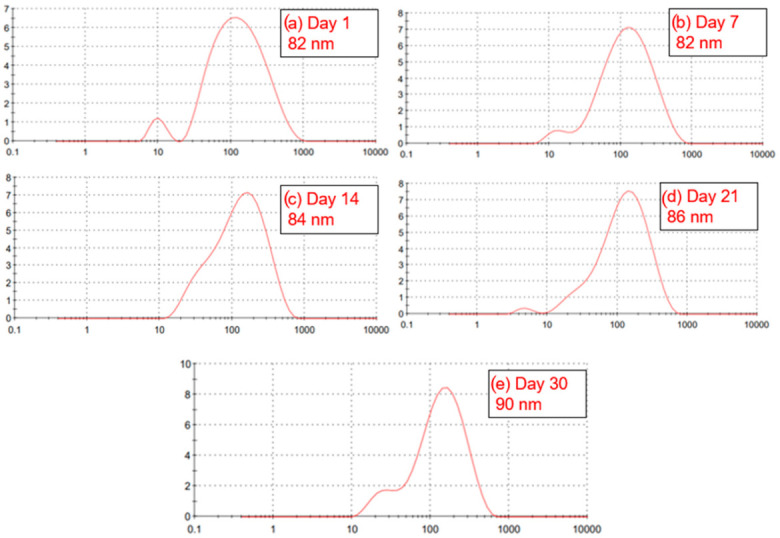
Droplet sizes obtained with an Ultrasonicator and their different sizes; (**a**) day 1; (**b**) day 7; (**c**) day 14; (**d**) day 21; (**e**) day 30.

In Figure 12 we have the graph of nanoemulsions obtained by Ultrasonicator had an initial average size of 82 nanometers on day 1, which remained unchanged until day 7, showing excellent stability in the first few days of storage. On day 14, there was a slight increase to 84 nanometers, followed by 86 nanometers on day 21, and finally, 90 nanometers on day 30. Over the 30 days, the total increase was only 8 nanometers, which represents a variation of less than 10% from the initial value. Similar variations can be found in studies using an Ultrasonicator that also managed to obtain low nanometer variations in a study using carvacrol. This subtle and progressive variation indicates that the system remained structurally stable, with minimal coalescence or droplet growth over time [69].

The stability observed in the first 7 days, with no change in droplet size, is especially important for commercial applications where the product may be stored for short periods before use. The gradual growth between day 14 and day 30 may be related to minor internal structural readjustments within the emulsion, such as interfacial relaxation, but it does not compromise its functionality. Internal rearrangements with minimal variations in droplet size have also been observed in Pickering-type nanoemulsions [70].

The nanoemulsion with a 2% (*w*/*w*) oil content showed minimal variation in droplet size throughout the entire storage period, indicating good physical stability under these conditions. The particle size remained stable at 130 nm on day 1 and day 7, with a slight increase to 131 nm on day 14 before returning to 130 nm on day 21 and 129 nm on day 30.

This suggests that this concentration was sufficient to maintain the structural integrity of the system, with the nanoemulsion size remaining virtually unchanged after one month. On the other hand, the formulation containing a 6% (*w*/*w*) oil phase showed an increase in droplet size over time. The particle size began at 79 nm on day 1, slightly increased to 81 nm on day 7, remained at 80 nm on day 14 and day 21, and ended at 83 nm on day 30. This variation was only about 5%, which is not a significant change. From the data, we can verify the particle growth kinetics as described by the Smoluchowski theory. The results showed a growth rate of approximately 0.10 nm per day, indicating that nanoemulsion has good kinetic stability, with a predicted slight and gradual growth over time [71].

### 3.9. pH Determination Results

The initial pH values for the high-pressure experiments ranged from 4.26 to 4.29, with a predominant value of 4.28, which is a slightly acidic formulation as expected for systems containing essential oil and chitosan [72]. This initial acidity is beneficial for food preservation by inhibiting microbial growth [73]. After 30 days, the final pH values showed very little variation, with a maximum registered value of 4.33. The overall change was within a small range of just 0.07 pH units, demonstrating the formulation’s good chemical stability over time. The slight pH increase in some samples is likely due to the degradation of volatile compounds, a common occurrence in mildly acidic aqueous systems. However, since these changes are minimal, they do not compromise the system’s function, and the pH remains within the ideal range for use in antimicrobial coatings [74].

The pH values for the nanoemulsions prepared using the Ultrasonication method were generally higher than those obtained by High-pressure homogenization. Initially, the pH ranged from 4.51 to 4.57, and after 30 days of storage, they reached a maximum of 4.63 in Experiment 11. The greater dispersion from this method might expose more functional groups of the chitosan, influence the medium’s ionic balance and cause a slight increase in the pH. Despite this difference, the values remained within the slightly acidic range considered ideal for antimicrobial systems [75].

## 4. Conclusions

Based on the findings of this study, we successfully analyzed the compounds in thyme essential oil, extracted chitosan from shrimp waste, and developed chitosan nanoparticles to stabilize nanoemulsions. We created a nanoemulsion formulation with proven stability over a four-month period, maintaining key physicochemical characteristics such as particle and droplet size, pH, and visual appearance. The extended stability of this system suggests that combining chitosan nanoparticles and essential oil with efficient emulsification methods, like High-pressure homogenization and an Ultrasonicator, is a promising strategy for preserving fruits and other perishable foods. Both methods were effective in creating stable nanoemulsions with nanometer-scale particles, though they showed important differences. Ultrasonication promoted greater component dispersion, which was reflected in particle size values and possibly a more efficient interaction between the aqueous and oil phases, thus improving system stability. High-pressure homogenization also proved its capability to produce nanoemulsions at the nanoscale. The 30-day stability assessment confirmed that the nanoemulsion system maintained its physicochemical characteristics with only minimal variations in droplet size and pH. Future studies may extend this approach to other polysaccharide-based systems to evaluate its applicability and performance.

## Figures and Tables

**Table 1:** Experimental variables and factor codification.

Type	Factor	Variable	Levels
Numeric	X_1_	Dispersed phase (g)	1.17–6.83
Numeric	X_2_	Aqueous phase (g)	93.17–98.83
Categorical	Block	Method	1 = Ultrasonication; 2 = High pressure

**Table 2:** Experiments performed according to the central composite design (CCD).

Run	Method	X_1_	X_2_	Dispersed Phase (g)	Aqueous Phase (g)
1	2	0	0	4.00	96.00
2	2	0	0	4.00	96.00
3	2	+α	0	6.83	96.00
4	2	−α	0	1.17	96.00
5	2	0	0	4.00	96.00
6	2	0	+α	4.00	98.83
7	2	0	−α	4.00	93.17
8	1	−1	+1	2.00	98.00
9	1	0	0	4.00	96.00
10	1	+1	+1	6.00	98.00
11	1	+1	−1	6.00	94.00
12	1	0	0	4.00	96.00
13	1	0	0	4.00	96.00
14	1	−1	−1	2.00	94.00

**Table 3:** Chemical Composition of *Thymus vulgaris* L. Essential Oil Identified by GC-MS.

Compound	Retention Time (min)	Relative Area (%)
Thymol	30.404	33.15808856
p-Cymene	17.165	18.15034676
Linalool	21.171	12.94962793
γ-Terpinene	18.902	6.849733018
Linalil acetate	28.341	6.772354266
α-Terpineol	25.673	3.769713935
Isoborneol	24.054	3.750203577
Caryophyllene	36.605	3.343934964
Carvacrol	30.708	3.023357
Eucaliptol	17.584	2.152167876
Alfa Pineno	12.434	1.689218951
D—Limoneno	17.390	0.665381996
(+)—2—Bornanone	23.240	0.665934741
Camphene	13.217	0.604656115
Isoterpinoleno	25.917	0.516375197
Terpinoleno	20.252	0.506678938
Isotujol	23.487	0.275979049
Endo-borneol	24.495	0.286805155
o-Cymene	16.918	0.179332556
Cis-verbenol	27.781	0.179582573
Isofenchol	21.731	0.178973248
1-Terpineol	22.856	0.168303506
Citral	29.184	0.163250083

**Table 4:** Results of Nanoemulsion, Droplet Size.

Run	Method	Average Droplet Size (nm)
1	High Pressure	105
2	High Pressure	106
3	High Pressure	70
4	High Pressure	130
5	High Pressure	108
6	High Pressure	106
7	High Pressure	90
8	Sonicator	130
9	Sonicator	87
10	Sonicator	77
11	Sonicator	79
12	Sonicator	82
13	Sonicator	85
14	Sonicator	126

**Table 5:** ANOVA of the factors influencing particle size.

Source	DF	Adj SS	Adj MS	F-Value	*p*-Value
Method	1	630.2	630.21	21.12	0.002
Dispersed phase	4	4889.0	1222.26	40.97	<0.001
Error	8	238.7	29.83		
Total	13	5299.2			

**Table 6:** Regression coefficients for droplet size as a function of method and oil phase concentration.

Term	Coefficient	SE Coefficient	t-Value	*p*-Value	VIF
Constant	99.97	1.93	51.70	<0.001	
Method	−9.17	1.99	−4.60	0.002	1.87
OF 1.7%	20.87	5.02	4.16	0.003	1.69
OF 2.0%	37.20	4.13	9.01	<0.001	1.68
OF 4.0%	−6.13	2.48	−2.48	0.038	1.13
OF 6.0%	−12.80	4.13	−3.10	0.015	1.68

**Table 7:** Model of R2.

S	R-Squared	Adjusted R-Squared
5.46199	95.50%	92.68%

**Table 8:** Test of Tukey.

Method	*n*	Mean (±SD)
High-pressure	7	109.133 ^a^
Ultrasonicator	7	90.800 ^b^

Note: Different uppercase letters indicate significant differences between oil phase concentrations (*p* < 0.05).

**Table 9:** Tukey test comparing the effects of the disperse phase.

OF (%)	n	Mean (±SD)
2.0	2	137.167 ^A^
1.7	1	120.833 ^A^
4.0	8	93.833 ^B^
6.0	2	87.167 ^BC^
6.8	1	60.833 ^C^

Note: Different uppercase letters indicate significant differences between oil phase concentrations (*p* < 0.05).

**Table 10:** MANOVA results in the effects of oil phase concentration and methods.

Factor	Criterion	Statistic	F-Value	Numerator DF	Denominator DF	*p*-Value
Oil Phase	Wilks’ Lambda	0.04654	40.970	4	8	<0.001
	Lawley-Hotelling	20.48484	40.970	4	8	<0.001
	Pillai’s Trace	0.95346	40.970	4	8	<0.001
	Roy’s Largest Root	20.48484	40.970	4	8	<0.001
Method	Wilks’ Lambda	0.27468	21.124	1	8	0.002
	Lawley-Hotelling	2.64054	21.124	1	8	0.002
	Pillai’s Trace	0.72532	21.124	1	8	0.002
	Roy’s Largest Root	2.64054	21.124	1	8	0.002

## Data Availability

The original contributions presented in this study are included in the article. Further inquiries can be directed to the corresponding authors.
